# Dual-Mode Manipulating Multicenter Photoluminescence in a Single-Phased Ba_9_Lu_2_Si_6_O_24_:Bi^3+^, Eu^3+^ Phosphor to Realize White Light/Tunable Emissions

**DOI:** 10.1038/s41598-017-15903-7

**Published:** 2017-11-21

**Authors:** Yue Guo, Sung Heum Park, Byung Chun Choi, Jung Hyun Jeong, Jung Hwan Kim

**Affiliations:** 1 Department of Physics, Pukyong National University, Busan, 608-737 South Korea; 2 Department of Physics, Dongeui University, Busan, 614-714 South Korea

## Abstract

A Bi^3+^ and Eu^3+^ ion co-doped Ba_9_Lu_2_Si_6_O_24_ single-phased phosphor was synthesized successfully via a conventional high-temperature solid-state reaction. X-ray diffraction, crystal structure analysis, diffuse reflectance and luminescent spectra, quantum efficiency measurements, and thermal stability analysis were applied to investigate the phase, structure, luminescent and thermal stability properties. From the analyses of the crystal structure and luminescent spectra, we observed four discernible Bi^3+^ luminescent centers with peaks at ~363.3, ~403.1, ~437.7, and ~494.5 nm. Moreover, due to the complex energy transfer processes among these Bi^3+^ centers, their relative emission intensity tightly depended on the incident excitation wavelength. Interestingly, the as-prepared phosphor could generate warm white light/tunable emission by changing the concentration of Eu^3+^ ions or adjusting the excitation wavelength. The energy transfer mechanism from Bi^3+^ to Eu^3+^ was confirmed via an electric dipole-dipole interaction, the energy transfer efficiencies $({\eta }_{T})$ from Bi^3+^ to Eu^3+^ were 50.84% and 40.17% monitoring at 410 and 485 nm, respectively. The internal quantum efficiency of the optimized Ba_9_Lu_2_Si_6_O_24_:Bi^3+^, Eu^3+^ phosphor was calculated to be 42.6%. In addition, the configurational coordinate model was carried out to explain the energy decrease of the phonon-electron coupling effect.

## Introduction

Recently, phosphor materials have attained great achievement and progress in various fields, including solid-state lighting, optical temperature sensors, flat panel displays, solar cells, and optical biomarkers^1–6^. As next-generation lighting devices, phosphor-converted white light-emitting diodes (w-LEDs) have received much more attention since w-LEDs provide extraordinary superiorities, such as low electric consumption, high electro-optical conversion efficiency, high brightness, good stability, fast response, and environmental friendliness^7–10^. Until now, combining blue-emitting InGaN-based LED chips and yellow-emitting Y_3_Al_5_O_12_:Ce^3+^ phosphors to make white light emission is one of the simplest and most efficient ways for commercial application^11,12^. However, such a combination shows a poor color rendering index (CRI < 80) and a high correlated color temperature (CCT > 4500 K) due to the lack of red-light contribution, which limits vivid applications^13,14^. To overcome the above mentioned problems, another improvement method is to employ near ultraviolet (near-UV) emitting LED chips (300–410 nm) coated with trichromatic phosphors. Unfortunately, the trichromatic phosphor system produces several inevitable problems, including complex coating, fluorescence reabsorption between different components, and non-uniformity of the luminescence properties, resulting in the degradation of luminous efficiency, increased manufacturing costs and a time-dependent shift of the color point^15,16^. To circumvent these drawbacks, a single-phased phosphor, which is fabricated by co-doping the sensitizer and activator ions into an appropriate host, with white light emission for near-UV pumped w-LEDs would be favorable alternative^17^.

Silicate materials are inexpensive and readily available, and silicate-based phosphor materials serve as promising luminescent materials in the use of phosphor-converted w-LEDs because of their structural diversity, relatively easy preparation, good thermal stability, and visible light transparency^18,19^. Recently, a silicate-based phosphor Ba_9_Lu_2_Si_6_O_24_ having an orthosilicate structure doped with various rare-earth ions has been extensively studied due to its favorable luminescence properties. For example, Liu *et al*. reported the luminescence properties of Eu^2+^-doped Ba_9_Lu_2_Si_6_O_24_ blue phosphor^20^, Liu *et al*. investigated the high thermal and radiation stability from Ce^3+^ doped Ba_9_Lu_2_Si_6_O_24_ green phosphor^7^; Song *et al*. studied energy transfer in Ce^3+^, Mn^2+^ co-doped Ba_9_Lu_2_Si_6_O_24_ red phosphor^19^. It should be noted that the silicate-based phosphor Ba_9_Lu_2_Si_6_O_24_ possesses multiple luminescent centers, as it contains three types of Ba centers and one type of Lu center. Therefore, multicentered materials doped with appropriate rare-earth ion have greater potential than that of a single-center material using proper center engineering^21^.

Among various RE ion activators, bismuth is typical ns^2^-type luminescent center, which is sensitive to the surrounding crystal field. It can emit UV, visible and near-infrared spectral regions attributing to its 6s6p-6s^2^ transition^22,23^. Thus far, the Bi^3+^ ion usually acts as an efficient sensitizer for Eu^3+^ ions in a large number of hosts^11,24^. In this work, we studied the multicentered photoluminescence characteristics and site engineering in a Bi^3+^ and Eu^2+^ co-doped Ba_9_Lu_2_Si_6_O_24_ phosphor to realize white light/tunable emission for phosphor-converted w-LEDs. Due to the complex energy transfer processes, the tuning of multicentered photoluminescence can be achieved by adjusting the excitation wavelength or controlling the rare-earth ion concentration.

## Results

### TG-DTA and Crystal Structure Analysis of Ba_9_Lu_2–*x*–*y*_Si_6_O_24_:*x*Bi^3+^, *y*Eu^3+^ (abbreviated as BLSO:*x*Bi^3+^, *y*Eu^3+^)

**Figure 1:**
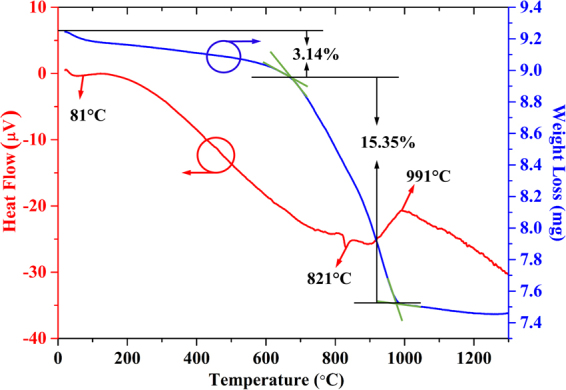
TG-DTA curves of the as-prepared blank BLSO precursor sample.

Figure 1 shows the TG-DTA curves of the as-prepared blank Ba_9_Lu_2_Si_6_O_24_ (abbreviated as BLSO) precursor sample, carried out to investigate the decomposition and thermal interaction processes among all the raw materials. It can be seen from the TG curve (blue curve) that the weight loss can be divided into two temperature regions: one is from room temperature to 630 °C with 3.14% weight loss, and the other is from 630 °C to 980 °C with 15.35% weight loss. The first weight loss is partially derived from the evaporation of residual water and ethanol contents in the powdered surface with an endothermic band at approximately 81 °C. Besides, the weight loss between 100 °C and 630 °C arises from the evaporation of remaining water molecules and decomposition of organic material. From the analysis, the second weight loss may be attributed to the decomposition of BaCO_3_ to CO_2_ gas following the equation:

1 $$BaC{O}_{3}\mathop{\to }\limits^{{\rm{\Delta }}}BaO+C{O}_{2}\uparrow $$

The experimental value of the weight loss is 15.35% with the above reaction. Simultaneously, we calculated the theoretical value according to equation (1) and the stoichiometric ratio, which is about 15.62%. The results show that both the theoretical and the experimental values are close to each other, confirming our hypothesis for the second weight loss process. In addition, BaCO_3_ possesses three crystallographic structures: γ-rhombohedral, β-hexagonal and α-cubic. One can see that the phase transformations in BaCO_3_ can be observed in the DTA curve (red curve). The endothermic peak at 821 °C and exothermic peak at 991 °C correspond to the phase transformations of γ → β and β → α, respectively. Above 980 °C, the slope of the TG curve gradually stabilizes, forming the BLSO host lattice.

**Figure 2:**
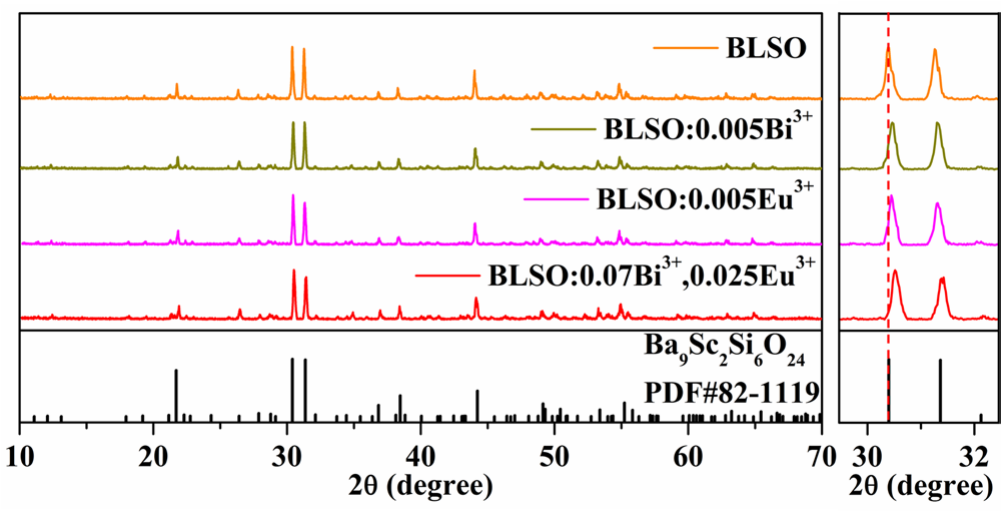
The XRD patterns of blank BLSO, BLSO:0.005Bi^3+^, BLSO:0.005Eu^3+^, and BLSO:0.07Bi^3+^,0.025Eu^3+^ phosphors and the standard pattern (PDF#82–1119).

Figure 2 depicts the XRD patterns of the blank BLSO, BLSO:0.005Bi^3+^, BLSO:0.005Eu^3+^ and BLSO:0.07Bi^3+^, 0.025Eu^3+^ phosphors, the standard pattern Ba_9_Sc_2_Si_6_O_24_ (PDF#82-1119) is shown as a reference. It can be clearly seen from the XRD analysis that all the diffraction peaks in these obtained solid solutions agree well with the standard pattern of Ba_9_Sc_2_Si_6_O_24_ (PDF#82-1119), which indicates that these obtained solid solutions have a single-crystal phase, and no impurities or secondary phases were found. In addition, doping trace amounts of Bi^3+^ and Eu^3+^ ions did not cause any notable change in the crystal lattice. From the enlarged image of Fig. 2, the two main diffraction peaks at 30.40° and 31.34° (2θ) shift slightly to higher angles as Bi^3+^ and Eu^3+^ are doped into the host. This observation results from lattice shrinkage induced by the substitution of the smaller Bi^3+^ and Eu^3+^ ions for the larger Ba^2+^ or La^3+^ ions.

**Figure 3:**
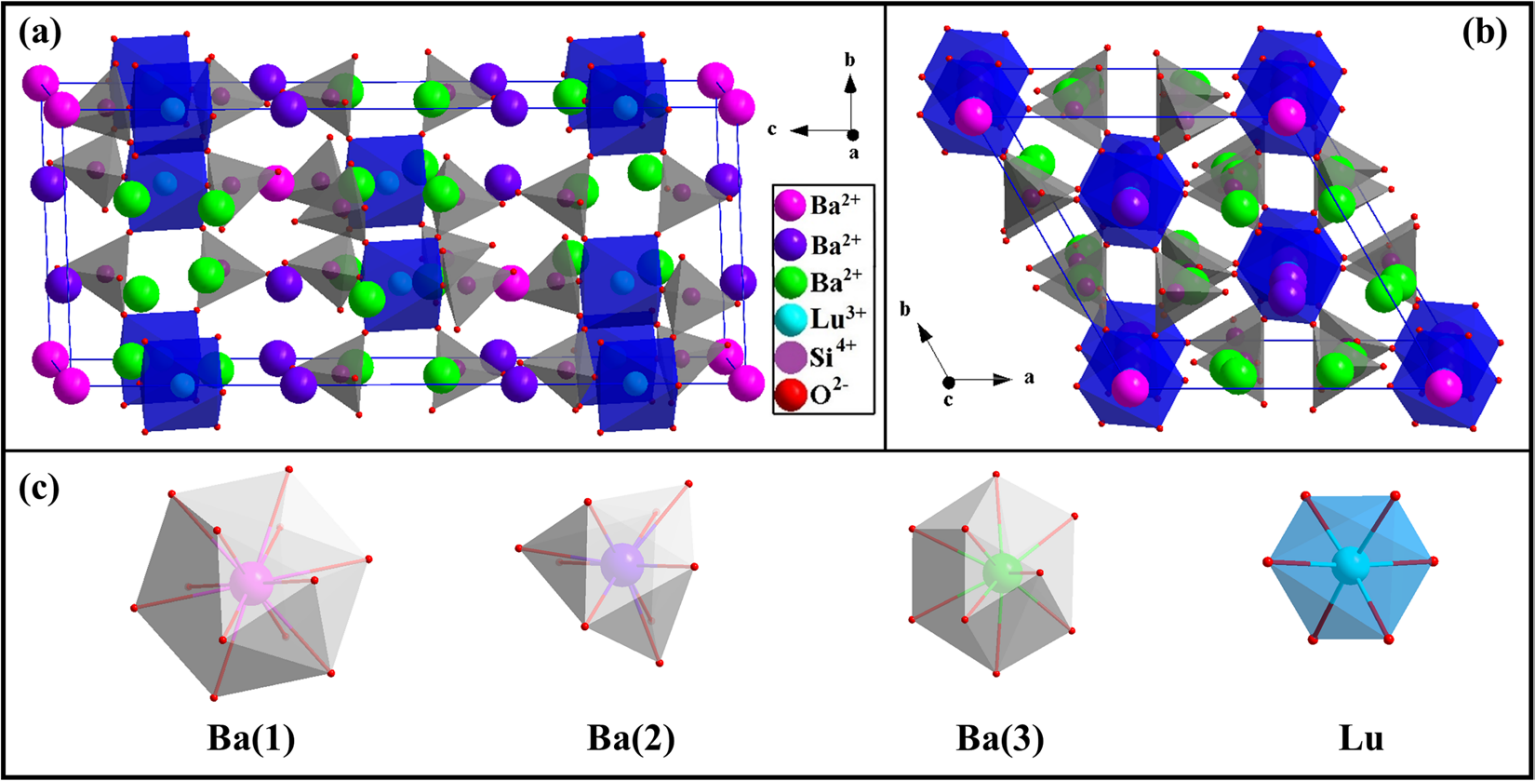
Crystal structure of BLSO unit cell viewed from the a-axis (**a**) and c-axis (**b**). The coordination environments of Ba(1), Ba(2), Ba(3) and Lu sites (**c**).

BLSO crystallizes in a rhombohedral structure and belongs to the $R\bar{3}$ (148) space group^20^. The crystal structure of the BLSO unit cell viewed from the a-axis and the c-axis is shown in Fig. 3. It exhibits the layered distribution in which the smaller tetrahedral SiO_4_ units and the larger octahedral LuO_6_ units are corner-shared as SiO_4_-LuO_6_-SiO_4_-LuO_6_, forming a rigid three-dimensional network^7^. In the crystal structure, the Lu^3+^ ions are located at the 6*c* Wyckoff position with 6-fold coordination, evolving a distorted LuO_6_ octahedron that has two different Lu-O bond lengths^20^. While the Ba^2+^ ions are designated in three independent sites: Ba(1) at the 3*a* Wyckoff position with 12-fold coordination, Ba(2) at the 6*c* Wyckoff position with 9-fold coordination and Ba(3) at the 18 *f* Wyckoff position with 10-fold coordination, forming three different distorted polyhedra with different Ba-O bond lengths. For easy distinguish, we can define the three distinct types of Ba sites and one Lu site as Ba(1), Ba(2), Ba(3) and Lu, respectively, and all these sites can be substituted by Bi^3+^ or Eu^3+^ ions. For the Ba atom sites, such as the 12-fold coordination Ba(3) site, Bi^3+^ (r = 1.45 Å) and Eu^3+^ (r = 1.23 Å) ions are smaller than Ba^2+^ (r = 1.61 Å) ions, and therefore, the Bi^3+^ and Eu^3+^ ions are expected to randomly substitute the Ba^2+^ sites. This result can be extended to the other Ba^2+^ ions. However, for the Lu atom site with 6-fold coordination, Bi^3+^ (r = 1.02 Å) and Eu^3+^ (r = 0.947 Å) ions are bigger than Lu^3+^ (r = 0.861 Å) ions. According to Vegard’s law, a complete solid solution should form if the size difference of ions is in the range of ±15%^25^. Therefore, Bi^3+^ and Eu^3+^ ions can also randomly substitute the Lu site.

### Luminescence Properties of Bi^3+^ Single-Doped Ba_9_Lu_2_Si_6_O_24_ Phosphor

**Figure 4:**
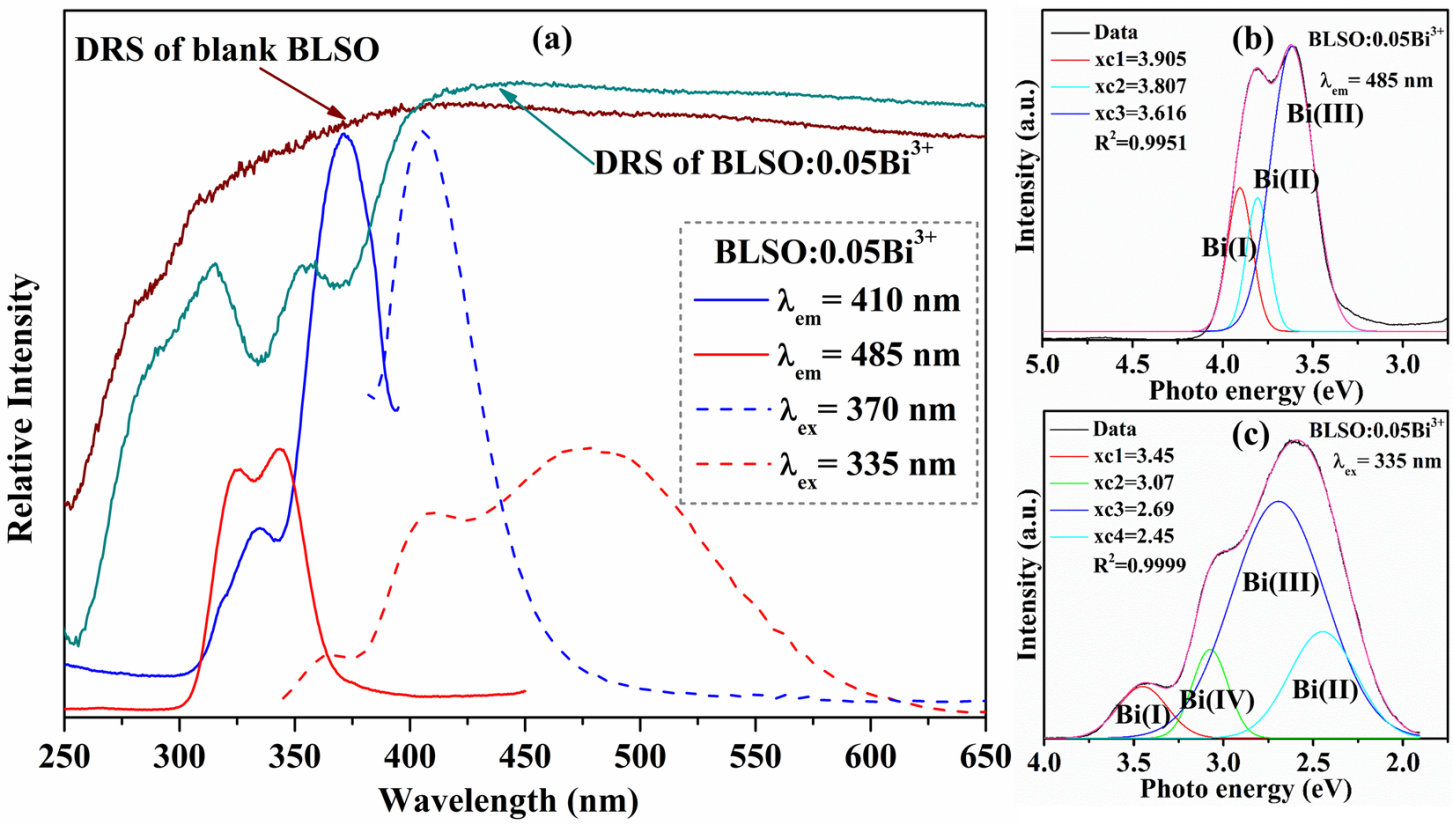
(**a**) Photoluminescence and excitation spectra of BLSO:0.05Bi^3+^ sample (λ_em_ = 410 nm and λ_em_ = 485 nm for excitation spectra, λ_ex_ = 335 nm and λ_ex_ = 370 nm for emission spectra); UV-vis DRS of blank BLSO and BLSO:0.05Bi^3+^ samples. (**b**) The Gaussian deconvolution of the excitation spectrum (λ_em_ = 485 nm). (**c**) The Gaussian deconvolution of the emission spectrum (λ_ex_ = 335 nm).

Upon different excitation wavelengths, the Bi^3+^ single-doped BLSO sample shows different luminescence features. Figure 4a shows the photoluminescence and excitation spectra of the BLSO: 0.05Bi^3+^ sample excited at 335/370 nm and monitored at 410/485 nm, respectively. There is no luminescence from blank BLSO, and thus, the observed different luminescence features should come from the Bi^3+^ ions. Under excitation of 370 nm, the BLSO:0.05Bi^3+^ sample exhibits a symmetric broad blue emission band ranging from 380 to 500 nm with a maximum value at 410 nm. Monitoring the emission at 410 nm produces the excitation spectrum, which consists of two broad absorption bands with peaks at around 335 and 370 nm. Upon 335 nm excitation, BLSO:0.05Bi^3+^ presents a different emission band ranging from 350 to 650 nm with three bands peaking at about 365, 410 and 485 nm. Figure 4b shows the excitation spectrum monitored at 485 nm, which can be deconvoluted into three symmetric absorption bands centered at 3.905 eV (317.5 nm), 3.807 eV (325.7 nm), and 3.616 eV (342.9 nm). Meanwhile, Fig. 4c shows the emission spectrum excited at 355 nm, which can be deconvoluted into four symmetric absorption bands centered at 3.45 eV (359.4 nm), 3.07 eV (403.9 nm), 2.69 eV (461.0) and 2.45 eV (506.1 nm). From this spectrum, we can conclude that there are at least four kinds of Bi^3+^ luminescent centers in the BLSO host. To simplify the discussion below, these four deconvoluted bands are denoted as Bi(I), Bi(II), Bi(III), and Bi(IV). According to previous studies and discussions, it is deduced that the blue emission band marked as Bi(IV) at ~ 410 nm is derived from Bi^3+^ ions substituted at the Lu^3+^ sites, while the remaining three emission bands result from Bi^3+^ ions substituted at the three distinct types of Ba sites. However, this is still unclear and requires additional study on how to assign the emission band to a particular Ba crystallographic site.

**Figure 5:**
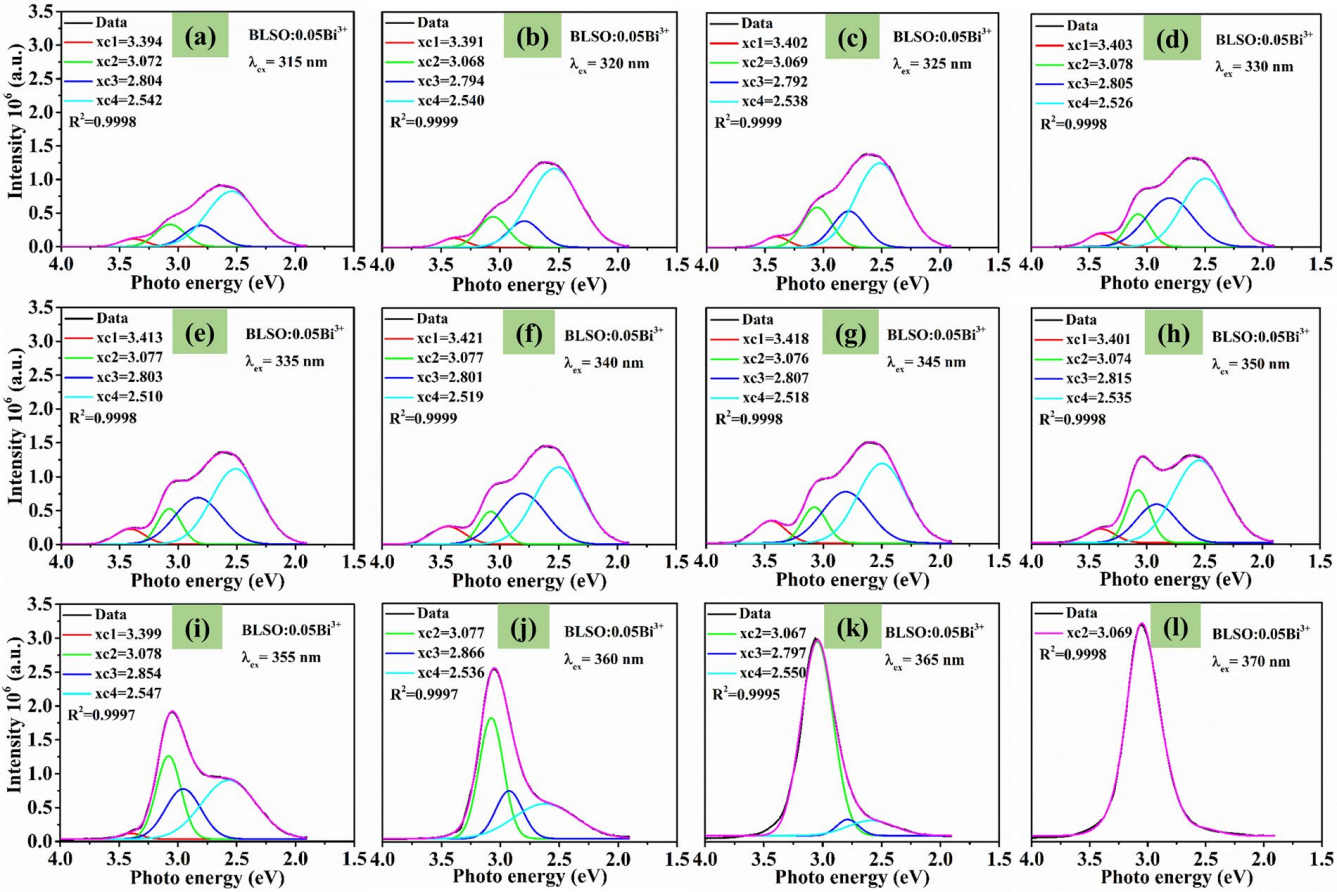
The Gaussian deconvolution for the emission spectra of BLSO:0.05Bi^3+^ sample under continuous excitation wavelengths from 315 to 370 nm with a step of 5 nm.

To investigate the energy transfer among the four kinds of Bi^3+^ ions, Fig. 5 shows the Gaussian deconvolution of the emission spectra for the BLSO:0.05Bi^3+^ sample under continuous excitation wavelengths from 315 to 370 nm with a step of 5 nm. From these figures, we can see that the intensity changes intuitively. As the excitation wavelength increases from 315 to 355 nm, there are always four emission bands with peaks at ~ 363.3 nm (3.413 eV), ~ 403.1 nm (3.077 eV), ~ 437.7 nm (2.833 eV), and ~ 494.5 nm (2.518 eV). However, as the excitation wavelength continues to increase from 360 to 370 nm, only one emission band of Bi(IV) remains. Based on these phenomena, it is reasonable that energy transfer occurs from Bi(I), Bi(II) and Bi(III) to Bi(IV). Furthermore, this energy transfer process is more efficient with an increasing excitation wavelength since the intensity of the Bi(IV) emission considerably increases. This may be due to the better energy match between the excitation wavelength with Bi(IV). On the other hand, it can be seen that energy transfer also occurred among the Bi(I), Bi(II) and Bi(III) ions since the three emission bands are observed under continuous excitation wavelengths from 315 to 355 nm.

The Commission International de L’Eclairage (CIE) chromaticity coordinates of the BLSO:0.05Bi^3+^ sample under continuous excitation wavelengths from 315 to 370 nm with a step of 5 nm are shown in Fig. S1. The inset lists the related calculated CIE chromaticity coordinates. It is clearly seen that the emission color varied from blue to bluish green by adjusting the excitation source from 315 to 340 nm with the CIE coordinates changing from (0.1721, 0.2268) to (0.1800, 0.2636). Nevertheless, the emission color shows a dramatic change as the excitation source increases from 315 to 340 nm, moving from bluish green to blue with the CIE coordinates largely varying from (0.1795, 0.2596) to (0.1950, 0.0968). Accordingly, the obtained phosphor can be used as a blue-emitting phosphor for w-LED applications. To obtain warm white light emission, we attempted to co-doped Eu^3+^ ions in the BLSO:Bi^3+^ system, as Bi^3+^ ions can be an efficient sensitizer for Eu^3+^ ions in various host lattices.

### Energy Transfer from Bi^3+^ to Eu^3+^ in the Ba_9_Lu_2_Si_6_O_24_ Phosphor with White Light/Tunable Emission

Figure S2 exhibits the photoluminescence and excitation spectra of the BLSO:0.005Eu^3+^ sample excited at 393 nm and monitored at 611 nm. The excitation spectrum shows a charge-transfer band (CTB) at 200 to 280 nm, originating from Eu^3+^-O^2−^, and a series of sharp lines located at 300 ~ 500 nm, resulting from the 4f-4f transitions of the Eu^3+^ ions. Upon excitation at 393 nm, the emission spectrum shows the typical ^5^D_0_ → ^7^F_J_ (J = 0, 1, 2, 3, 4) transition of the Eu^3+^ ions in the range of 550–700 nm. It should be noted that ^5^D_0_ → ^7^F_1_ with ∆*J* = 1 and ^5^D_0_ → ^7^F_2_ with ∆*J* = 2 belong to the magnetic-dipole and electric-dipole transitions, respectively. The intensity of ^5^D_0_ → ^7^F_2_ is extraordinarily strong as compared to the ^5^D_0_ → ^7^F_1_ transition, which indicates that the Eu^3+^ ions are located in asymmetric sites in the BLSO host. It is interesting to note that the combination of these Eu^3+^ emission lines with the Bi^3+^ emission in the BLSO host is helpful to generate white light performance.

**Figure 6:**
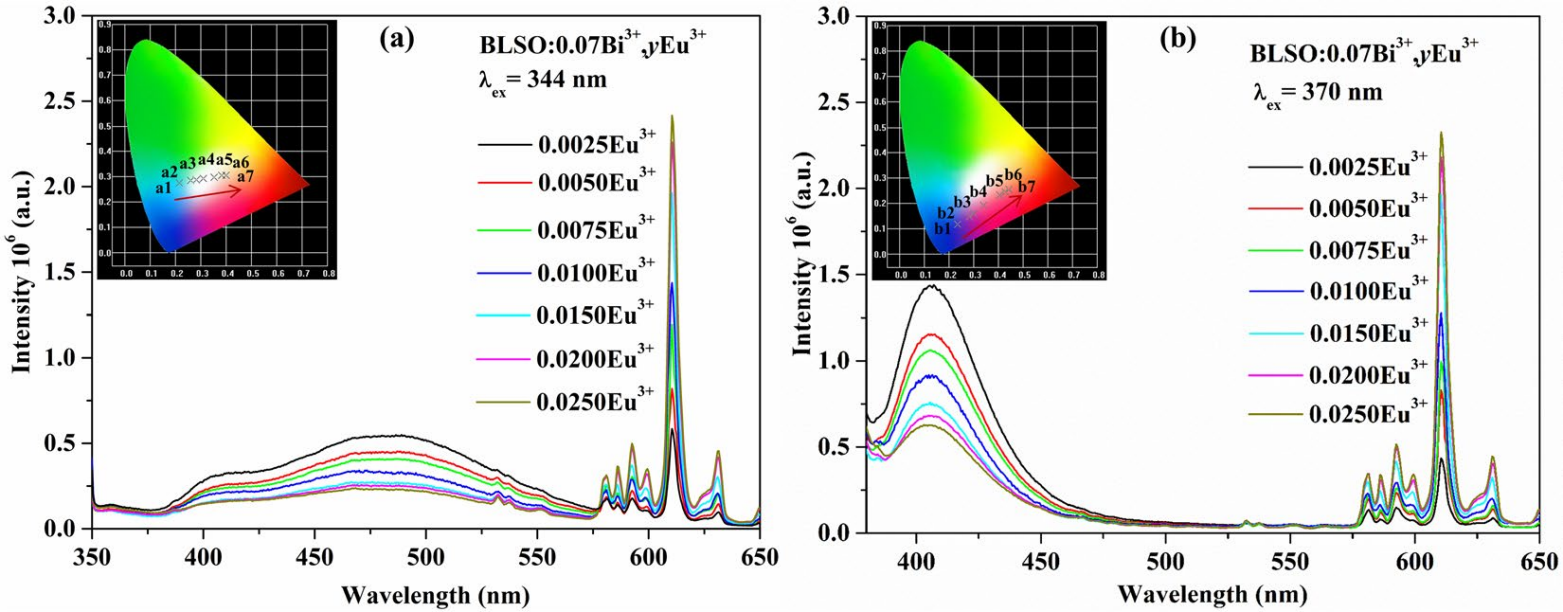
Emission spectra of BLSO:0.07Bi^3+^, *y*Eu^3+^ (*y* = 0.0025–0.0250) samples excited at 344 nm (**a**) and 370 nm (**b**). The inset shows the related CIE chromaticity diagram.

Comparing the emission spectrum of BLSO:0.05Bi^3+^ with the excitation spectrum of BLSO:0.005Eu^3+^ in Fig. 4a and Fig. S2, there is a significant spectral overlap in the range of 350–500 nm. According to this phenomenon, there may exist energy transfer from Bi^3+^ to Eu^3+^ in the BLSO host. In order to study the energy transfer from the Bi^3+^ to Eu^3+^ ions, the emission spectra of the BLSO:0.07Bi^3+^, yEu^3+^ (y = 0.0025–0.0250) phosphors excited at 344 and 370 nm are shown in Fig. 6a,b, respectively. Obviously, at either excitation wavelength, as the Eu^3+^ concentration increases, the emission intensity of Bi^3+^ rapidly reduces, while the emission intensity of Eu^3+^ gradually increases. In addition, there is no Eu^3+^ concentration quenching as *y* increases from 0.0025 to 0.025. Based on this, we can clearly deduce the existence of energy transfer from Bi^3+^ to Eu^3+^ in the BLSO host. The related CIE chromaticity diagram of BLSO:0.07Bi^3+^, *y*Eu^3+^ (*y* = 0.0025–0.0200) samples is listed in the inset of Fig. 6. It is found that the location of the color coordinates (*x*, *y*) changes from bluish green to pink across the entire white region under 344 nm excitation and from blue to pink under 370 nm excitation. Therefore, it is possible to tune the emission color by changing the excitation wavelength and adjusting the Bi^3+^/Eu^3+^ molar ratio. Table S1 displays the calculated CIE chromaticity coordinates and correlated color temperatures (***T***_***cct***_) of the BLSO:0.07Bi^3+^, *y*Eu^3+^ (*y* = 0.0025–0.0200) samples excited at 344 and 370 nm. By comparison, the color coordinate point of a5 (x = 0.3523, y = 0.2973) is particularly close to the National Television Standards Committee (NTSC) white point (x = 0.330, y = 0.330) with a ***T***_***cct***_ value at 4571 K. Consequently, these results demonstrated that the combination of these Eu^3+^ emission lines with Bi^3+^ emission in the BLSO host could generate warm white emission.

In general, the energy transfer mechanism from the sensitizer to the activator takes place at a high doping concentration by an exchange interaction or a multipolar interaction. According to Dexter’s energy transfer proposal and Reisfeld’s approximation of the multipolar interaction, the following formula can be obtained^26,27^:

2 $$\frac{{\eta }_{0}}{\eta }\approx \frac{{I}_{0}}{I}\propto {C}^{n/3}$$

where, ${\eta }_{0}$ and $\eta$ are the energy transfer efficiencies of the sensitizer (Bi^3+^) in the absence and presence of activator (Eu^3+^), respectively. Similarly, *I*_0_ and *I* are the luminescent intensities of Bi^3+^ in the absence and presence of Eu^3+^ ions, respectively. Normally, the values (${\eta }_{0}/\eta$) can be estimated from the related luminescence intensity, *I*_0_ */I*. *C* is the total concentration of the Bi^3+^ and Eu^3+^ ions; n = 6, 8 and 10 correspond to the dipole-dipole, dipole-quadrupole and quadrupole-quadrupole interactions, respectively. In this case, the *I*_0_ */I* versus *C* ^n/3^ plots with linear fittings are illustrated in Fig. S3, and the R^2^ values are observed from the relationships when n = 6, 8, and 10. The best fitting is R^2^ = 99.98% when n = 6. Therefore, the dipole-dipole interactions dominate energy transfer from Bi^3+^ to Eu^3+^ in the BLSO host.

**Figure 7:**
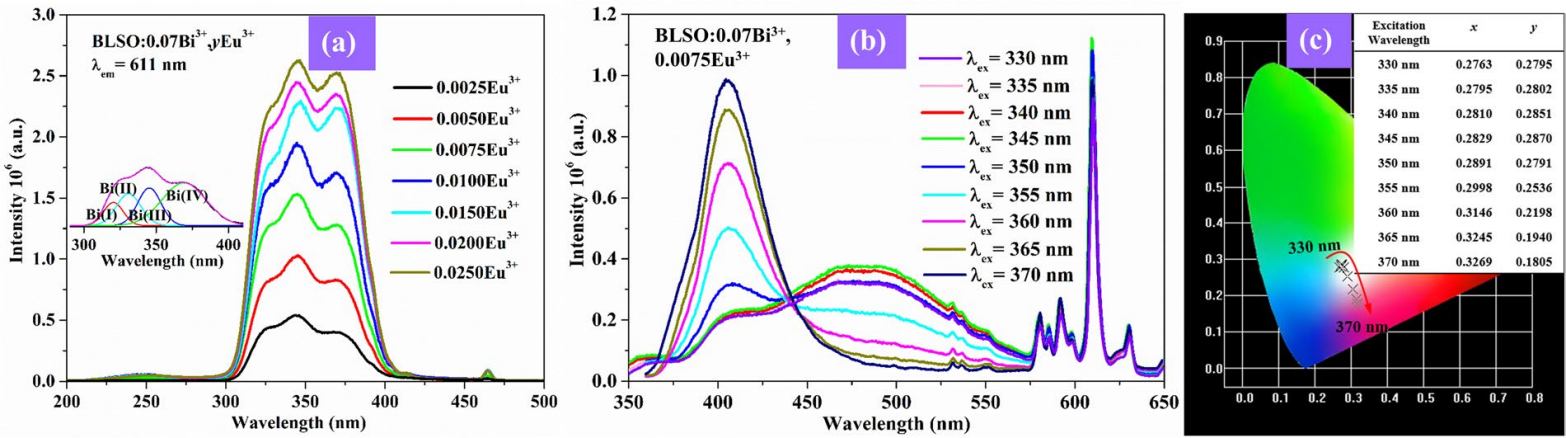
(**a**) Excitation spectra of BLSO:0.07Bi^3+^, *y*Eu^3+^ (*y* = 0.0025–0.0250) monitored at 611 nm. Inset shows the Gaussian deconvolution for broad band at 300–400 nm. (**b**) Emission spectra of BLSO:0.07Bi^3+^, 0.0075Eu^3+^ under continuous excitation wavelengths from 330 to 370 nm with a step of 5 nm. (**c**) The related CIE chromaticity coordinates of BLSO:0.07Bi^3+^, 0.0075Eu^3+^.

To determine which Bi^3+^ centers make contributions to the emission of the Eu^3+^ ions, Fig. 7a exhibits the excitation spectra of the BLSO:0.07Bi^3+^, *y*Eu^3+^ (*y* = 0.0025–0.0250) samples monitored at 611 nm. In addition to the Eu^3+^-O^2−^ CTB at 200–280 nm and the ^7^F_0_ → ^5^D_2_ transition of the Eu^3+^ ions at 464 nm, the broad band at 300–400 nm originates from the Bi^3+^ ions. From the Gaussian deconvolution of this broad band, it is clearly to seen that there are four small Gaussian peaks fitted. By comparison of Fig. 7a with Fig. 4a, these four Gaussian peaks match well with the Bi(I), Bi(II), Bi(III), and Bi(IV) bands, which demonstrate that these four kinds of Bi^3+^ centers contribute to the Eu^3+^ emission. To more intuitively explain the contribution of the various Bi^3+^ centers to Eu^3+^ emission, the emission spectra of the BLSO:0.07Bi^3+^, 0.0075Eu^3+^ sample under continuous excitation wavelengths from 330 to 370 nm with a step of 5 nm are displayed in Fig. 7b. We attempted to excite various Bi^3+^ centers with the different excitation sources, and the emission spectra show a series of Bi^3+^ and Eu^3+^ emissions with different intensities and different emission colors. The emission color is tunable from white to reddish purple with the excitation wavelength increasing from 330 to 370 nm, as shown in Fig. 7c. In conclusion, we developed two simple strategies to obtained white light/tunable emission: one is to change the concentration of Eu^3+^ ions in the BLSO:0.07Bi^3+^ phosphor, and the other is to adjust the excitation wavelength.

In general, energy transfer from Bi^3+^ to Eu^3+^ can also be demonstrated by an investigation of the dynamic luminescence process. The decay curves of the BLSO:0.07Bi^3+^, yEu^3+^ (*y* = 0.0025–0.0250) samples excited at 355 nm and monitored at 410 and 485 nm were compared and are presented in Fig. S4. According to the fitting analyses, the decay curves of the Bi^3+^ emission at 410 and 485 nm are non-exponential, indicating that there is more than one relaxation process. The average lifetimes *τ*_*avg*_ of these samples can be estimated using the simple equation^28^:

3 $${\tau }_{avg}=\frac{{{\int }^{}}_{0}^{\infty }I(t)t{\rm{dt}}}{{{\int }^{}}_{0}^{\infty }I(t)}$$

where *I(t)* is the luminous intensity at time *t*. According to equation (3), the average lifetimes of Bi^3+^ were evaluated and listed in Table S2. The lifetimes of Bi^3+^ decrease monotonically with an increasing Eu^3+^ ion concentrations, strongly supporting energy transfer from Bi^3+^ to Eu^3+^.

Using these lifetimes, the energy transfer efficiencies (${\eta }_{T}$) from Bi^3+^ to Eu^3+^ can be approximately calculated by an equation defined by Paulose *et al*.^29^:

4 $${\eta }_{T}=1-\frac{{\tau }_{S}}{{\tau }_{{S}_{0}}}$$

where *τ*_*S*0_ and *τ*_*S*_ are the intrinsic lifetime of the sensitizer (Bi^3+^) and the lifetime of the sensitizer (Bi^3+^) in the presence of the activator (Eu^3+^), respectively. Using this equation, the ${\eta }_{T}$ values from Bi^3+^ to Eu^3+^ monitoring at 410 nm emission are calculated to be 12.35%, 21.89%, 28.39%, 32.88%, 42.09%, and 50.84%, while those monitoring at 485 nm emission are 4.26%, 8.11%, 14.70%, 20.29%, 29.25%, and 40.17%.

**Figure 8:**
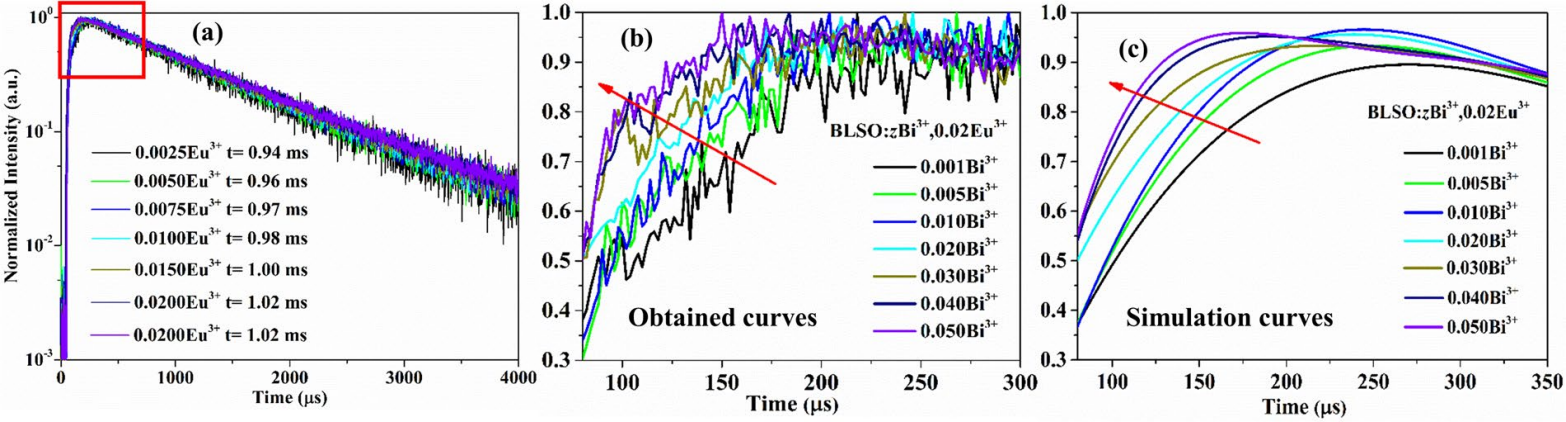
(**a**) Decay curves of BLSO:zBi^3+^, 0.02Eu^3+^ samples excited at 370 nm and monitored at 611 nm. The obtained curves of enlarged initial rise-up region from 80 to 300 μs (**b**) and the corresponding simulation curves (**c**).

Figure 8a illustrates the decay curves of the BLSO:zBi^3+^, 0.02Eu^3+^ samples excited at 370 nm and monitored at 611 nm. As shown in Fig. 8a, there are two different processes: one is a rise-up process, and the other is a decay process. In the initial rise-up process, energy is trapped by the Bi(4) center and then transfers to the Eu^3+^ ions. Figure 8b,c show the obtained curves of the enlarged initial rise-up region from 80 to 300 μs and the corresponding simulation curves, respectively. One can see that the rise-up process shows a high-speed rising due to the increasing Bi^3+^ content, which indicates that energy transfer from Bi^3+^ to Eu^3+^ becomes more efficient. For the decay process, the decay curves show single-exponential decay behavior following the equation:

5 $$I(t)={I}_{0}+A\exp (-t/\tau )$$

where *I*(*t*) and *I*_0_ are the luminescence intensities at time *t* and *t* = 0, respectively; *τ* is the luminescent lifetime; *A* is a constant. According to equation (5), the lifetimes of Eu^3+^ are calculated to be 0.94, 0.96, 0.97, 0.98, 1.00, 1.02, and 1.02 ms with z = 0.001, 0.005, 0.01, 0.02, 0.03, 0.04, and 0.05, respectively. In this case, the luminescent lifetime of the activator (Eu^3+^) increases since the Eu^3+^ ion emission does not show concentration quenching.

**Figure 9:**
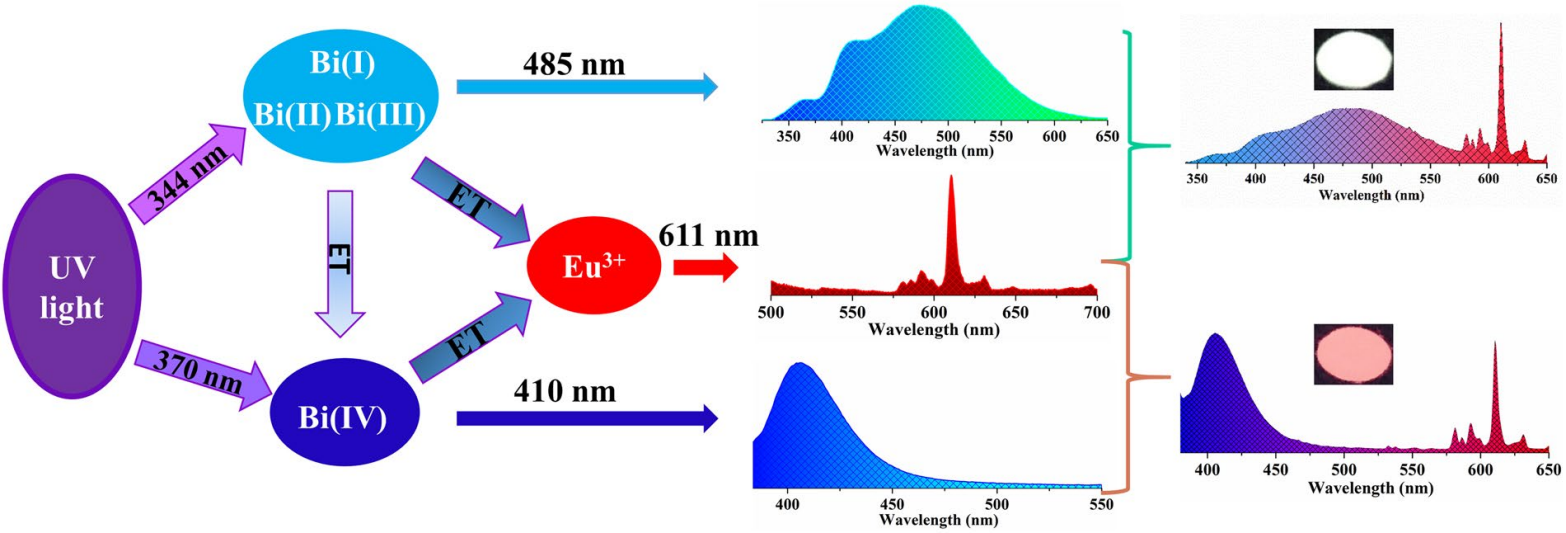
Energy transfer scheme of generating white light/tunable emissions in Bi^3+^ and Eu^3+^ co-doped BLSO phosphor upon UV light excitation.

Based on the luminescence and decay behaviors given in Figs 4, 6, 7, and S4, the energy transfer scheme of generating white light/tunable emission in the Bi^3+^ and Eu^3+^ co-doped BLSO phosphor upon UV light excitation is presented in Fig. 9. Under excitation of 344 nm, the Bi(I), Bi(II) and Bi(III) centers are excited by UV light and then give a broad blue/green emission band. Simultaneously, due to energy transfer from these three Bi^3+^ centers to the Eu^3+^ center, warm white light emission can be obtained from the Bi^3+^ and Eu^3+^ co-doped BLSO phosphor. In addition, under 370 nm excitation, energy is trapped by the Bi(IV) center, giving a strong blue emission. At the same time, by effective energy transfer from the Bi(IV) center to the Eu^3+^ center, a tunable emission phosphor from the blue to red region is achieved.

### Thermal Analysis of the Ba_9_Lu_1.92_Si_6_O_24_:0.07Bi^3+^, 0.0075Eu^3+^ Phosphor

**Figure 10:**
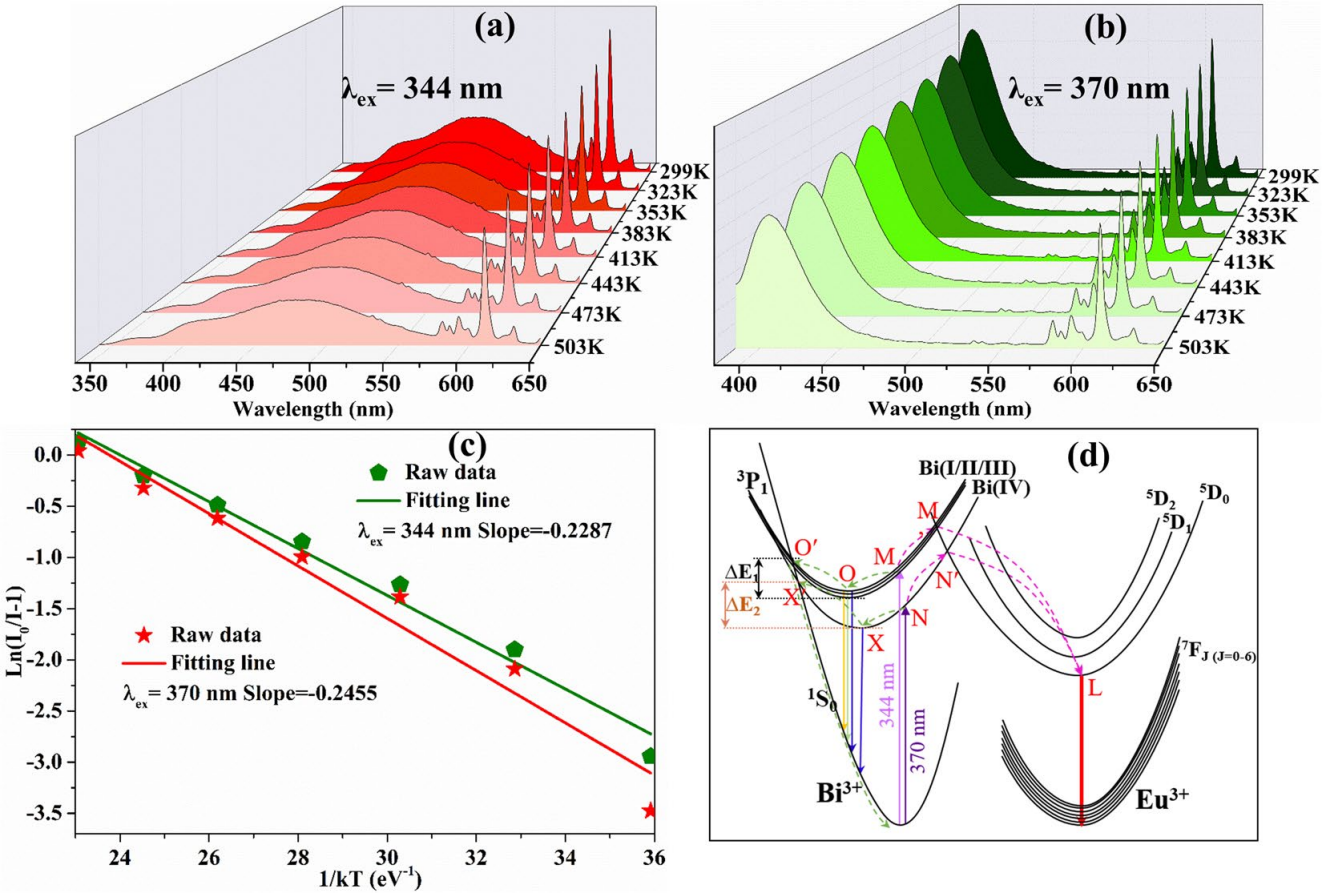
Temperature-dependent luminescence properties of BLSO: 0.07Bi^3+^,0.0075 Eu^3+^ phosphor along with temperature increase from 299 to 503 K under 344 nm (**a**) and 370 nm (**b**). (**c**) The plots of ln[*I*_0_ */I*−1] against 1/*k*T. (**d**) The configurational coordinate diagram of the ground and excited states of Bi^3+^ and Eu^3+^.

It is accepted that the thermal stability of a phosphor is of great significance for its practical applications, including the color rendering index and the light output of w-LEDs^30^. Therefore, Fig. 10a,b show the temperature-dependent luminescence properties of the BLSO: 0.07Bi^3+^, 0.0075Eu^3+^ phosphor upon 344 and 370 nm excitations, respectively, with an increasing temperature from 299 to 503 K. We can observe that both emission intensities of the BLSO:0.07Bi^3+^, 0.0075Eu^3+^ sample with an increasing temperature gradually decline. Moreover, the emission intensities at 423 K (150 °C) of the BLSO:0.07Bi^3+^, 0.0075Eu^3+^ sample under 344 and 370 nm excitations remain at approximately 66.1% and 72.8% of their initial values, respectively. To meet application requirements, the thermal stability of the obtained phosphor must be strengthened. Generally, strengthening of the thermal stability can be achieved by optimizing the preparation conditions to reduce vacancies and improve crystallinity. It is well known that the activation energy (*E*_a_) is a good indicator to assess the thermal stability of phosphors^31^. It can be calculated from the Arrhenius equation^32^:

6 $$In\{\frac{{I}_{0}}{I}-1\}=InA-\frac{{E}_{a}}{kT}$$

where *I*_0_ and *I* are the integrated intensity at room temperature and a particular operating temperatures, respectively; *E*_*a*_ and T are the calculated activation energy and the operating temperature (K), respectively; A and *k* are a constant for a certain host and the Boltzmann constant (8.62 × 10^−5^ eV K^−1^), respectively. As depicted in Fig. 10c, through a best-fitting of the ln[*I*_*0*_ */I*−1] against 1/*kT* plot using equation (6), the calculated activation energies *E*_*a*_ are 0.2287 and 0.2455 eV for 344 and 370 nm excitations, respectively. From the above investigation, we can see that the thermal stability of the obtained phosphor excited at 370 nm is more stable than that excited at 344 nm, which is consistent with the calculated results.

The configurational coordinate diagrams of the ground and excited states of Bi^3+^ and Eu^3+^ as well as the CTB of Eu^3+^-O^2–^ are displayed in Fig. 10d. To simplify the structure, we divided the bismuth ions into two types: one for Bi(I), Bi(II) and Bi(III) centers excited at 344 nm; the other for Bi(IV) center excited at 370 nm. Both excitations show similar spectroscopic and energy transfer properties, and thus, the following description is focused on one of them, namely, the 344 nm excitation. Under excitation at 344 nm, the electrons of the Bi(I), Bi(II) and Bi(III) centers absorb the energy and are excited from the ^1^S_0_ ground state to the ^3^P_1_ excited state at room temperature. Then, some of the electrons undergo a non-radiative process, relaxing to the lowest state positions of ^3^P_1_ (point O), from where the electrons return to the ground state ^1^S_0_ by radiative transition. Some of the electrons cross point M’, resulting in energy transfer from the Bi^3+^ to Eu^3+^ ions and subsequent relaxation to the lowest state positions of ^5^D_0_, from where the electrons return to the ground state ^7^F_J_ (J = 0–6) by radiative transition. Furthermore, some of the electrons will donate their energy via energy transfer to the Bi(IV) ions. With an increasing temperature, the electrons at ^3^P_1_ might overcome ∆E_1_ with stronger phonon-electron coupling. Simultaneously, the electrons can be excited from point O to O’, from where the electrons undergo a non-radiative process relaxing to ^1^S_0_, leading to a rapid decrease of the number of electrons.

Another important indicator parameter for practical applications is the quantum efficiency (QE). The internal QE value can be calculated based on the following equation^33^:

7 $$\eta =\frac{{\int }^{}{L}_{s}}{{\int }^{}{E}_{R}-{\int }^{}{E}_{s}}$$

where *L*_*s*_ means the emission spectrum of the obtained sample, ${E}_{R}$ represents the spectrum of the excitation light from the empty integrated sphere (without the sample), and ${E}_{s}$ is the spectrum of the light used for exciting the sample. The internal QE of the BLSO:0.07Bi^3+^, *y*Eu^3+^ (*y* = 0.0025–0.0250) phosphors excited at 344 and 370 nm were calculated and listed in Table S3. The highest QE values reach 42.6% and 32.9% upon 344 and 370 nm excitation, respectively. Since the QE closely depends on the preparation conditions, particle size, defects, and morphology, it can be further improved by optimizing the synthesis process^26^.

## Discussion

In summary, we reported a systematic study on the Bi^3+^, Eu^3+^ co-doped BLSO single-phased phosphor with warm white light/tunable emission by manipulating energy transfer in multicentered photoluminescence. Upon different excitation wavelengths, the Bi^3+^ single-doped BLSO sample showed different luminescence. Four discernible Bi^3+^ (Bi(I)- Bi(IV)) luminescent centers were confirmed by analyzing the crystal structure and luminescent spectra. Energy transfer from Bi(I), Bi(II) and Bi(III) to Bi(IV) was manipulated by changing the excitation wavelength. For Bi^3+^, Eu^3+^ co-doped BLSO phosphor, two simple strategies were described to obtained white light/tunable emissions one was to change the concentration of Eu^3+^ ions in the BLSO:0.07Bi^3+^ phosphor, and the other was to adjust the excitation wavelength. The energy transfer mechanism from Bi^3+^ to Eu^3+^ in the BLSO host was dominated by dipole-dipole interactions, the energy transfer efficiencies (${\eta }_{T}$) from Bi^3+^ to Eu^3+^ were as high as 50.84% and 40.17% monitoring at 410 and 485 nm, respectively. In addition, as the temperature increased to 423 K (150 °C), the emission intensities of the BLSO:0.07Bi^3+^, 0.0075Eu^3+^ sample retained approximately 66.1% and 72.8% of their initial values under 344 and 370 nm excitations, respectively. All the results show that the BLSO: Bi^3+^, Eu^3+^ phosphor has potential application fin phosphor-converted w-LEDs.

## Methods

### Materials and Synthesis

These series of Ba_9_Lu_2–*x*_Si_6_O_24_:*x*Bi^3+^ (*x* = 0.005, 0.01, 0.02, 0.03, 0.04, 0.05, 0.06), Ba_9_Lu_*1*.*93*–*y*_Si_6_O_24_:0.07Bi^3+^,*y*Eu^3+^ (*y* = 0.0025, 0.0050, 0.0075, 0.0100, 0.0150, 0.0200, 0.0250) and Ba_9_Lu_*1*.*98*–*z*_Si_6_O_24_:zBi^3+^,0.02Eu^3+^ (*z* = 0.001, 0.005, 0.01, 0.02, 0.03, 0.04, 0.05) phosphors were prepared by the standard solid-state reaction. These phosphors were synthesized by mixing raw materials of BaCO_3_ (99.90%), Lu_2_O_3_ (99.99%), SiO_2_ (99.99%), Bi_2_O_3_ (99.99%), and Eu_2_O_3_ (99.99%) in air. All raw materials were commercially available and were used as received. Typically, all starting materials were weighed and mixed with each other, and then the mixture was ground about 40 min with proper ethanol addition in an agate mortar. After that, the mixture based precursors were pre-sintered at 850 °C for 3 h, which was followed by an intermediate grinding for 20 min to improve the homogeneity of these samples. Then, re-sintered at 1400 °C for 5 h and ground again for further characterization. This work was supplied by the Display and Lighting Phosphor Bank at Pukyong National University.

### Materials Characterization

The thermogravimetric-differential thermal analysis (TG-DTA) of blank Ba_9_Lu_2_Si_6_O_24_ powder were obtained by a Material Analysis and Characterization TG-DTA 2000 system with a heating rate of 10 °C/min from room temperature to 1300 °C in air. The phase purity and the crystalline structure were checked by the powder X-ray diffraction (XRD) analysis (Bruker D8 Advance) over the angular range 10° ≤ 2θ ≤ 70° with a step size of 0.02°, with Cu K_α_ irradiation (λ = 1.5406 Å) operating at 40 kV voltage and 40 mA current. Ultraviolet-visible diffuse reflectance spectra (UV-vis DRS) were performed on a V-670 UV-vis spectrophotometer (JASCO Corp., Japan). Photoluminescence excitation and emission spectra were measured using Photon Technology International (PTI, USA) fluorimeter equipped with a 60 W xenon lamp and the fluorescence lifetimes of Ba_9_Lu_*1*.*98*–*z*_Si_6_O_24_:zBi^3+^, 0.02Eu^3+^ (*z* = 0.001–0.07) samples were measured with a phosphorimeter attached to the fluorescence spectrophotometer with a 25 W xenon flash lamp. The fluorescence lifetimes of BLSO:0.07Bi^3+^, yEu^3+^ (*y* = 0.0025–0.0250) samples were measured under a 355 nm Nd:YAG laser excitation with a pulse duration of 5 ns. Temperature-dependent luminescence properties were obtained by a fluorescence spectrophotometer (SCINCO FS-2) with a heating apparatus as heating source. The photoluminescence quantum efficiency (QE) was carried out by a spectrofluorometer (JASCO, FP-8500, Japan) equipped with an integrating sphere attachment (ISF-834).

## Electronic supplementary material

Supplementary Information
