# Supplementary material for: Dual-Mode Manipulating Multicenter Photoluminescence in a Single-Phased Ba9Lu2Si6O24:Bi3+, Eu3+ Phosphor to Realize White Light/Tunable Emissions
Source: Sci Rep. 2017 Nov 21;7:15884. doi: 10.1038/s41598-017-15903-7 (PMC5698322; doi:10.1038/s41598-017-15903-7)
Supplement: Supplementary file 1 — Supplementary Information [file 41598_2017_15903_MOESM1_ESM.pdf]

Supplementary Information

Dual-Mode Manipulating Multicenter Photoluminescence in a Single-Phased $Ba_9Lu_2Si_6O_{24}:Bi^{3+},Eu^{3+}$ Phosphor to Realize White Light/Tunable Emissions

Yue Guo[1], Sung Heum Park[1], Byung Chun Choi[1], Jung Hyun Jeong[1,*], Jung Hwan Kim[2]

[1]*Department of Physics, Pukyong National University, Busan 608-737, South Korea*

[2]*Department of Physics, Dongeui University, Busan 614-714, South Korea*

[*]Corresponding author: Jung Hyun Jeong, E-mail: jhjeong@pknu.ac.kr

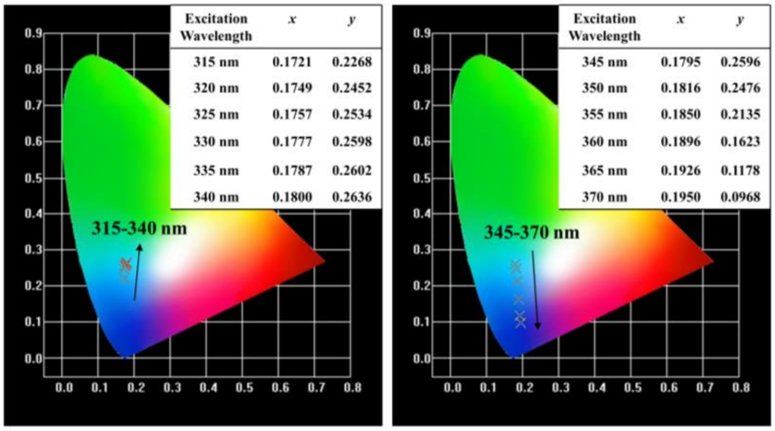

Figure S1 | The CIE chromaticity coordinates of BLSO:$0.05Bi^{3+}$ sample under continuous excitation wavelengths from 315 to 370 nm with a step of 5 nm. The inset lists the related calculated CIE chromaticity coordinate.

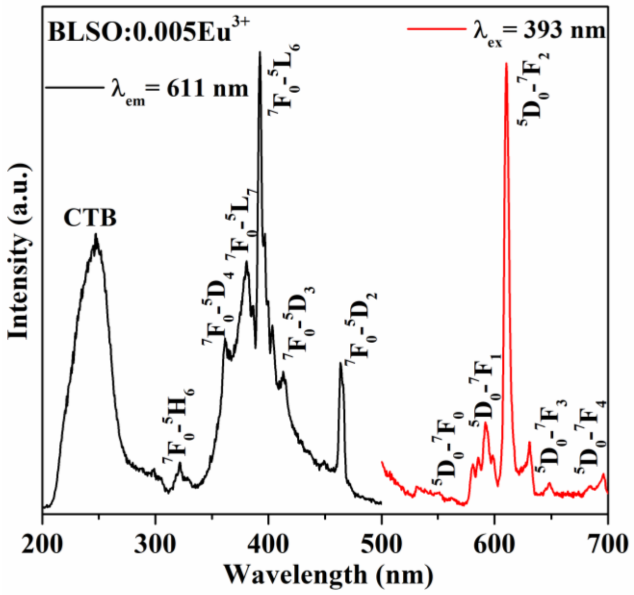

Figure S2 | Photoluminescence and excitation spectra of BLSO:0.005Eu^{3+} sample (λ_{em} = 611 nm for excitation spectrum, λ_{ex} = 393 nm for emission spectrum).

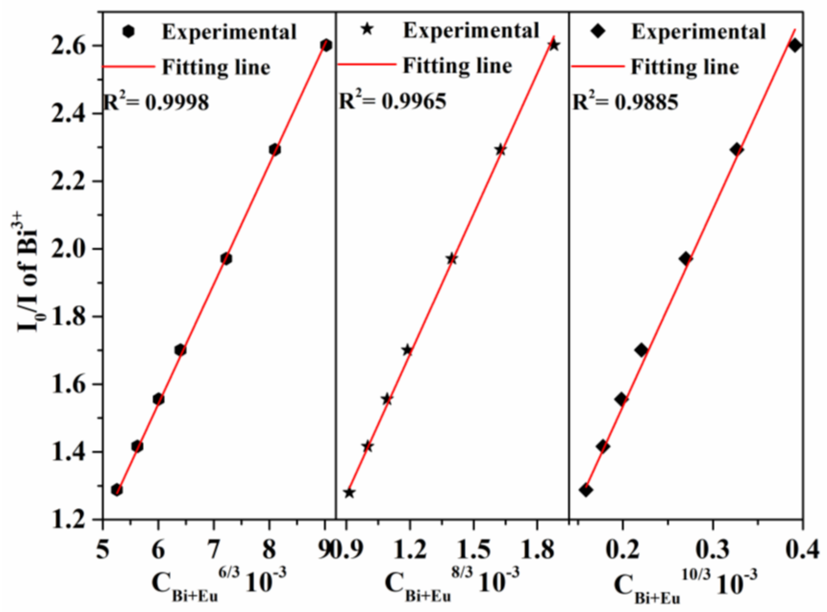

Figure S3 | Dependence of I_0/I on $C^{n/3}$ in BLSO:0.07Bi^{3+},$y$$Eu^{3+}$ (y = 0.0025-0.0250) phosphors.

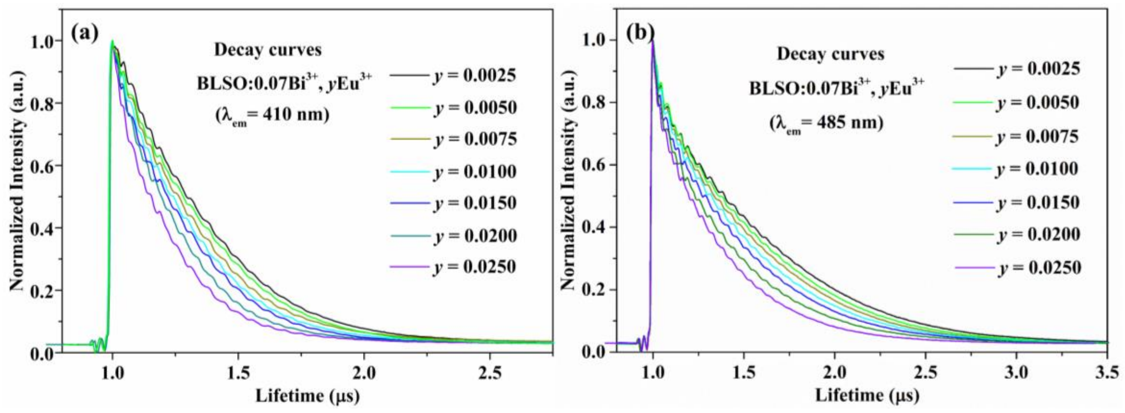

Figure S4 | Decay curves of BLSO:0.07Bi^{3+},yEu^{3+} (y = 0.0025-0.0250) samples monitored at 410 nm (a) and 485 nm (b).

Table S1 | Calculated CIE chromaticity coordinates and correlated color temperature (***T_{cct}***) of BLSO:0.07Bi^{3+},*y*Eu^{3+} (*y* = 0.0025-0.0200) samples excited at 344 and 370 nm.

BLSO:0.07Bi^{3+}, *y*Eu^{3+}	CIE (λ_{ex}=344 nm)	*Tcct*(K)	CIE (λ_{ex}=370 nm)	*Tcct*(K)
y= 0.0025	a1 (0.2154, 0.2741)	-	b1 (0.2351, 0.1170)	2531
y= 0.0050	a2 (0.2594, 0.2844)	-	b2 (0.2918, 0.1569)	2165
y= 0.0075	a3 (0.2863, 0.2843)	9843	b3 (0.3000, 0.1596)	1873
y= 0.0100	a4 (0.3207, 0.2814)	6298	b4 (0.3494, 0.2024)	2462
y= 0.0150	a5 (0.3523, 0.2973)	4571	b5 (0.4056, 0.2336)	4571
y= 0.0200	a6 (0.3822, 0.3040)	3098	b6 (0.4283, 0.2445)	5441
y= 0.0250	a7 (0.3996, 0.3046)	2551	b7 (0.4420, 0.2544)	4896

Table S2 The lifetimes of BLSO:0.07Bi^{3+},yEu^{3+} (*y* = 0.0025-0.0250) samples monitored at 410 and 485 nm.

**Sample**	**410 nm**	**485 nm**
**BLSO:0.07Bi^{3+},0.0025Eu^{3+}**	385.44 ns	628.48 ns
**BLSO:0.07Bi^{3+},0.0050Eu^{3+}**	337.82 ns	601.70 ns
**BLSO:0.07Bi^{3+},0.0075Eu^{3+}**	301.08 ns	577.53 ns
**BLSO:0.07Bi^{3+},0.0100Eu^{3+}**	276.01 ns	536.07 ns
**BLSO:0.07Bi^{3+},0.0150Eu^{3+}**	258.69 ns	500.94 ns
**BLSO:0.07Bi^{3+},0.0200Eu^{3+}**	223.22 ns	444.67 ns
**BLSO:0.07Bi^{3+},0.0250Eu^{3+}**	189.47 ns	376.01 ns

Table S3 The QE values of BLSO:0.07Bi^{3+},*y*Eu^{3+} (*y* = 0.0025, 0.0050, 0.0075, 0.0100, 0.0150, 0.0200, 0.0250) phosphors under 344 and 370 nm excitation.

**Sample**	**QE (%) (λ_{ex} = 344 nm)**	**QE (%) (λ_{ex} = 370 nm)**
BLSO:0.07Bi^{3+},0.0025Eu^{3+}	33.6	22.3
BLSO:0.07Bi^{3+},0.0050Eu^{3+}	31.1	25.3
BLSO:0.07Bi^{3+},0.0075Eu^{3+}	37.8	30.7
BLSO:0.07Bi^{3+},0.0100Eu^{3+}	42.6	32.9
BLSO:0.07Bi^{3+},0.0150Eu^{3+}	39.3	31.8
BLSO:0.07Bi^{3+},0.0200Eu^{3+}	36.7	27.6
BLSO:0.07Bi^{3+},0.0250Eu^{3+}	31.3	21.4
